# Exploring the relationship between temperament traits, psychological symptoms, and functional disability assessed with the WHODAS 2.0 in persons with multiple sclerosis

**DOI:** 10.3389/fneur.2025.1561995

**Published:** 2025-08-12

**Authors:** Carmenrita Infortuna, Maria Buccafusca, Anita Maria Stella Graceffa, Enrica Maiorana, Ray Wang, Siddarth Ganesh, Orion Yedidia, Antonio Bruno, Fiammetta Iannuzzo, Florian P. Thomas, Fortunato Battaglia

**Affiliations:** ^1^Department of Biomedical and Dental Sciences and Morphofunctional Imaging, Policlinico Universitario, University of Messina, Messina, Italy; ^2^Unit of Neurology and Neuromuscular Disorders, Department of Clinical and Experimental Medicine, University of Messina, Messina, Italy; ^3^Williams College, Williamstown, MA, United States; ^4^Department of Medical Sciences, Hackensack Meridian School of Medicine, Nutley, NJ, United States; ^5^Department of Neurology, Hackensack University Medical Center, Hackensack Meridian School of Medicine, Nutley, NJ, United States

**Keywords:** multiple sclerosis, WHODAS 2.0, temperament traits, anxiety, depression, stress

## Abstract

**Background:**

The relationship between psychological comorbidity and functional impairment in multiple sclerosis (MS) remains to be thoroughly investigated. This study examined the associations between temperament traits, psychological comorbidities, and disability as measured by the World Health Organization Disability Assessment Schedule (WHODAS) 2.0 in persons with Relapsing–Remitting Multiple Sclerosis (RRMS).

**Methods:**

In this cross-sectional study, persons with RRMS underwent a comprehensive assessment of temperament profiles, psychological status, and functional disability. Assessment tools included the Depression Anxiety Stress Scale (DASS-21) and the Temperament Evaluation of Memphis, Pisa, Paris, and San Diego Autoquestionnaire (TEMPS-A) short form. Functional status was evaluated using the 36-item WHODAS 2.0. Multivariate regression analyses were performed to evaluate relationships between variables.

**Results:**

The study cohort comprised 105 persons with RRMS Hierarchical regression models showed that age and disease duration were significant predictors, with age positively associated with D02 and D06 dimensions, and disease duration linked to D02, D05, and D06 WHODAS 2.0 dimensions. Among temperament traits, hyperthymic temperament showed negative associations across multiple dimensions. Anxiety had strong positive association with disability. Model fit improved significantly with each step, with Step 3 explaining additional variance.

**Conclusion:**

These findings demonstrate significant associations between temperamental characteristics, particularly hyperthymic traits, −anxiety, and functional disability in persons with RRMS. Future research should investigate these relationships over time to understand causal links and create better treatments to slow disability progression. These findings could help improve how we assess and treat patients.

## 1 Introduction

Multiple Sclerosis (MS) causes progressive neurological dysfunction through inflammation-mediated demyelination in the central nervous system. Persons with – Relapsing–Remitting Multiple Sclerosis (RRMS) often display multisystem deficits that impair functional capacity and impact their quality of life (1, 2). Thus, clinical assessment tools are essential for quantifying disease progression and functional impairment (3). In RRMS, the Expanded Disability Status Scale (EDSS) is a typical standardized assessment tool to quantify and track longitudinal changes across various neurological domains, such as pyramidal, cerebellar, brainstem, sensory, bowel/bladder, visual, and cognitive functions. Addressing the methodological limitations of the EDSS is crucial for improving patient care (4, 5). Given the subjectivity of clinical evaluation, there are concerns about the EDSS inter-rater reliability and its sensitivity in detecting slight changes, particularly in cognitive non-motor symptoms (6, 7). The Multiple Sclerosis Functional Composite (MSFC) was developed to address the limitations of traditional ordinal scales like the EDSS (8). This tool has proven effective in quantifying upper limb function, ambulation, and cognitive functions (8). However, the scale has significant limitations and requires proper training and a labor-intensive interpretation of results (*Z*-score) (9). More recently, the Multiple Sclerosis Performance Test (MSPT), a tablet-based assessment tool, has been introduced to evaluate multiple functional domains, including walking speed, manual dexterity, processing speed, contrast sensitivity, and patient-reported outcomes (10). Although the MSPT addresses several of the MSFC’s limitations, further validation studies are required to establish its reliability and clinical utility (11). Hence, current clinical tools for assessing MS disability have significant limitations. As our understanding of MS deepens and new therapies emerge, we need additional instruments to evaluate patients fully.

The World Health Organization Disability Assessment Schedule 2.0 (WHODAS 2.0) assesses functional disability across six key domains: understanding and communicating, mobility, self-care, getting along with people, life activities, and participation in society (12). The World Health Organization (WHO) developed the scale to keep with the International Classification of Functioning, Disability, and Health (ICF) framework parameters. This assessment tool is reliable and suitable across different cultures for clinical care and research (12). WHODAS 2.0 has been shown to be effective in evaluating disability in RRMS. However, the factors influencing functional disability in this group are not yet well-defined (13–17). Previous studies in patients with neurological and psychiatric disorders have confirmed a strong link between disability and psychological comorbidities, including anxiety, depression, and stress, highlighting the importance of investigating this association in RRMS (18–20). For instance, depression in RRMS can decrease motivation and interest in daily activities, increasing, in this way, perceived disability. Anxiety is often characterized by distress and fear, and prolonged stress can intensify physical symptoms and reduce coping abilities, further impacting disability (21). The connection between mental health and functional disability is further reinforced by evidence that personality traits, which increase susceptibility to psychiatric disorders, are linked to a reduced quality of life in persons with RRMS (22). Furthermore, there is a lack of research on the influence of temperament—innate, stable emotional traits that form the foundation of personality in persons with MS. Temperament traits, particularly those related to emotional reactivity, inhibitory control, and activity level, play a crucial role in psychiatric predisposition and reactivity throughout development. Temperament is defined as early-appearing, biologically rooted variations in emotional and behavioral responses, exhibiting moderate stability and influenced by genetic and environmental factors (23). Core dimensions such as activity level, mood, adaptability, and persistence have been linked to vulnerability for various psychiatric conditions including anxiety, depression, bipolar disorder, and substance use disorders (24). The Temperament Evaluation of Memphis, Pisa, Paris, and San Diego Autoquestionnaire (TEMPS-A) identifies five affective temperament types—cyclothymic, depressive, hyperthymic, irritable, and anxious—that serve as subsyndromal phenotypes predictive of mood disorder onset and severity (25). For instance, cyclothymic temperament, characterized by rapid mood shifts and affective instability, is strongly associated with bipolar spectrum disorders and heightened emotional reactivity to stressors (26), while anxious temperament correlates with increased stress sensitivity and anxiety disorders (27). Conversely, hyperthymic temperament, marked by elevated energy and resilience, may modulate mood regulation differently but still influence psychiatric outcomes under stress (28). These temperament traits interact dynamically with neurobiological systems—such as those governing impulsivity, sensation seeking, and inhibitory control—and are influenced by genes affecting synaptic plasticity and associative learning (29, 30). Emerging evidence suggests that, persons with MS score higher in depressive, cyclothymic, irritable, and anxious temperaments compared to healthy controls (31). Similarly, research based on Cloninger’s model suggests that people with MS show higher levels of harm avoidance and lower levels of reward dependence (32). Thus, it is conceivable that specific temperament traits heighten the vulnerability to mood and anxiety disorders in MS, which in turn exacerbate cognitive dysfunction and functional impairment, factors directly linked to disability measured by the WHODAS.

To address this gap in the literature, the present observational, cross-sectional study aims to investigate the following research questions: (1) to what extent do demographic variables and disease duration predict functional disability (WHODAS 2.0) in persons with RRMS? (2) Do temperament traits (TEMPS-A: cyclothymic, depressive, hyperthymic, irritable, and anxious) significantly improve the prediction of functional disability beyond demographic and disease-related factors? (3) Does the addition of psychological symptoms (DASS-21: depression, anxiety, and stress) provide significant incremental validity in predicting functional disability assesses with WHODAS 2.0 in persons with RRMS? We hypothesize that (1) demographic variables and longer disease duration will be associated with greater functional disability, (2) temperament traits will significantly improve disability prediction beyond Step 1 variables, (3) DASS-21 symptoms will provide significant incremental validity beyond Steps 1 and 2.

## 2 Methods

### 2.1 Participants

This observational, cross-sectional study was conducted at the University of Messina, Italy. A total of 105 participants with RRMS defined with McDonald criteria (2017 revision) (33) and with Lublin criteria (34) were recruited at the Multiple Sclerosis Center, Department of Neurology, from September 2023 to December 2023. All patients who were older than 18 years old and on disease-modifying therapy were informed about the study by their treating physician during their regular follow-up visits. We excluded individuals with a history of severe psychiatric and neurologic disorders, active exacerbation of MS requiring steroid treatment in the last 30 days before the enrollment into our study, or significant cognitive impairment. The Montreal Cognitive Assessment (MoCA) Italian version was used to evaluate cognitive function. The MoCA assesses various cognitive domains, including attention, executive function, memory, language, visuospatial skills, abstract thinking, calculation, and orientation. Higher scores indicate better performance (0–30 range). Based on a previous Italian validation study (35), a cut-off of 17.36 was recommended for screening purposes for detecting cognitive impairment in the Italian population. Nonetheless, in our study we have used a more conservative cut off and we have included in the study only subjects with a MoCA score > 24. The study was approved by the local IRB al performed following the Declaration of Helsinki. Written informed consent was obtained before inclusion.

### 2.2 Assessment

During a regular follow-up visit we assessed the participants’ sociodemographic characteristics (age, gender, education, living status). The educational level was categorized in 4 groups according to the years at school (not attended school, 0 years; Primary school, 5 years; Secondary school, 8 years; High school, 13 years; University degree ≥17 years). We then ascertained the date and type of diagnosis (RRMS).

The Temperament Evaluation of Memphis, Pisa, Paris, and San Diego Auto-questionnaire (TEMPS-A) short version was administered in its validated Italian form to assess affective temperaments. This 39-item self-report questionnaire evaluates five temperamental dimensions: depressive, cyclothymic, hyperthymic, irritable, and anxious. Higher scores indicate a stronger expression of that temperamental trait. The Italian version shows robust psychometric properties (36). In our study, the Cronbach’s *α* was >0.73 for each domain.

The DASS-21 is a commonly used self-report questionnaire that evaluates the intensity of depression, anxiety, and stress symptoms. It is a reliable and efficient measure of emotional well-being and has been validated across different populations. Depression subscale indicates feelings of sadness, hopelessness, worthlessness, detachment, disinterest, lack of pleasure, and sluggishness. The anxiety subscale measures symptoms associated with physical arousal, tension, situational stress, and anxious feelings. The stress subscale assesses difficulty in relaxing, nervousness, irritability, and impatience. Participants rate their symptoms on a Likert scale over the past week. Higher scores suggest more severe depression, anxiety, or stress. In this study, the Cronbach’s *α* for this questionnaire was >0.76 for each domain (37).

The WHODAS 2.0 is a 36-item assessment evaluating disability across diverse cultures in six areas: understanding and communicating (cognition), mobility, self-care, social relationships, daily activities, and community participation. Participants rate their task difficulties over the past 30 days from 1 (none) to 5 (extreme/unable). Examining specific activities provides detailed insights into daily functions. An item response theory algorithm converts responses to 0–100 scores, with higher numbers indicating greater disability. The assessment showed strong reliability in our study (Cronbach’s *α*: total score = 0.81, all dimensions > 7.4) (38).

### 2.3 Statistical analysis

Data (continuous and categorical variables) were reported as mean (standard deviation, SD) and (%). Descriptive statistics were calculated for all variables. We have used Cronbach’s alphas to assess the internal consistencies of the psychological variables (temperament traits, anxiety, depression, and stress) and the WHODAS 2.0 (total score and different dimensions). Next, we performed a multiple correlation analysis to assess whether the psychological variables were associated with disability. Furthermore, we employed hierarchical regression models to examine predictors of disability (WHODAS 2.0 total and subdomain scores) in three sequential steps: (1) demographic/clinical factors (sex, age, education, relationship status, disease duration), (2) temperament traits (TEMPS-A subscales), and (3) psychiatric symptoms (DASS-21 subscales), reporting standardized beta coefficients (β) with significance levels (*p < 0.05, **p < 0.01) and assessing model fit through *R*^2^ change (Δ*R*^2^) and *F*-tests, while controlling for multicollinearity All analyses were performed with SPSS Statistics, version 23 (IBM Corp., Armonk, NY, United States).

## 3 Results

The study included 105 person with RRMS, with a mean age of 48.4 years (SD = 15), predominantly female (70.5%, *n* = 74), and mostly partnered (78.1%, *n* = 82). Education levels were varied, with high school being the most common (48.6%, *n* = 51). The mean disease duration was 15 years (SD = 9.6), and temperament assessments (TEMPS-A) revealed higher scores for hyperthymic (0.5, SD = 0.2) and anxious (0.5, SD = 0.3) traits. Psychological symptoms (DASS-21) indicated moderate stress (6.7, SD = 4.7), depression (5.1, SD = 4.3), and anxiety (4.6, SD = 4.4), while disability assessments (WHODAS 2.0) showed significant challenges in participation in society (mean = 30, SD = 20.7) and mobility (mean = 27.6, SD = 26.8), with a total disability score of 21.7 (SD = 19), reflecting moderate overall disability in the study population. Data are reported in Table 1. The intercorrelation matrix results of the variables are shown in Table 2. The correlation analysis revealed significant associations between clinical variables and the total WHODAS 2.0 disability score in persons with RRMS. Psychological distress (DASS-21) showed strong positive correlations, with stress (*r* = 0.381, *p* < 0.001), anxiety (*r* = 0.526, *p* < 0.001), and depression (*r* = 0.372, *p* < 0.001) all linked to higher overall disability. Temperament traits (TEMPS-A) also played a role, with cyclothymic (*r* = 0.262, *p* = 0.007), depressive (*r* = 0.295, *p* = 0.002), and anxious (*r* = 0.245, *p* = 0.012) temperaments positively associated with greater disability. Conversely, hyperthymic temperament correlated negatively (*r* = −0.319, *p* = 0.001), suggesting a protective effect. Age and disease duration did not significantly correlate with total disability (*p* > 0.05). We performed seven hierarchical multiple linear regression models employing as dependent variables the WHODAS 2.0 dimensions score (D01–D06) and the total sum score. The predictors were entered in steps: step 1 (age, sex, education, relationship, and disease duration), step 2 (temperament dimensions scores), and step 3 (DASS-21-dimension). Model 7 (WHODAS Total Score) demonstrated a progressive increase in explanatory power across the three steps. The initial Step 1 was non-significant [*R*^2^ = 0.038, *F*_(5,99)_ = 0.782, *p* = 0.565], with no individual predictors reaching significance. The addition of affective temperaments in Step 2 substantially improved model fit [Δ*R*^2^ = 0.217, total *R*^2^ = 0.255, *F*_(10,94)_ = 3.211, *p* = 0.001], with hyperthymic temperament as significant predictor (*β* = −0.350, *p* = 0.001), indicating a strong protective effect against overall functional disability. The final model incorporating DASS-21 scales further enhanced predictive validity [Δ*R*^2^ = 0.125, total *R*^2^ = 0.379, *F*_(13,91)_ = 4.278, *p* < 0.001], with both hyperthymic temperament (*β* = −0.257, *p* = 0.010) and anxiety (*β* = 0.409, *p* = 0.006) emerging as significant predictors, demonstrating opposing effects on functional disability.

**Table 1:** Demographic, and clinical outcome measures of the study population.

IO	Mean (SD)	*N*	%
Age (years)	48.4 (15)		
Gender			
Female		74	70.5
Male		31	29.5
Education			
Primary school, 5 years		0	0
Secondary school, 8 years		33	31.4
High school, 13 years		51	48.6
University degree ≥17		21	20
Relationship			
Single		23	21.9
Partnered		82	78.1
Disease duration (years)	15(9.6)		
TEMPS-A			
Cyclothymic	0.4(0.3)		
Depressive	0.2(0.2)		
Irritable	0.1(0.1)		
Hyperthymic	0.5(0.2)		
Anxious	0.5(0.3)		
DASS-21			
Depression	5.1(4.3)		
Anxiety	4.6(4.4)		
Stress	6.7(4.7)		
WHODAS 2.0			
Understanding and communicating	22(22.3)		
Getting around	27.6(26.8)		
Self-care	11.4(20.5)		
Getting along with people	13.3(20)		
Life activities	26 (25.5)		
Participation in society	30(20.7)		
Total disability score	21.7(19)		

TEMPS-A, temperament evaluation of memphis, Pisa, Paris and San Diego-autoquestionnaire short version; DASS-21, Depression-Anxiety-Stress Scale-21; WHODAS 2.0, World Health Organization Disability Assessment Schedule.

**Table 2:** Correlation of clinical variables with WHODAS 2.0 disability scores.

		1	2	3	4	5	6	7	8	9	10	11	12	13	14	15	16	17
1. Age		–																
	*p*	–																
2. Disease duration		0.630^**^	–															
	*p*	0.000	–															
3. TEMPS-A Cyclothymic		−0.207^*^	−0.165	–														
	*p*	0.034	0.093	–														
4. TEMPS-A Depressive		−0.093	−0.184	0.360^**^	–													
	*p*	0.343	0.061	0.000	–													
5. TEMPS-A Irritable		−0.254^**^	−0.159	0.440^**^	0.394^**^	–												
	*p*	0.009	0.106	0.000	0.000	–												
6. TEMPS-A hyperthymic		−0.116	−0.148	−0.211^*^	−0.094	0.113	–											
	*p*	0.240	0.131	0.031	0.340	0.252	–											
7. TEMPS-A Anxious		0.030	−0.089	0.491^**^	0.365^**^	0.173	0.020	–										
	*p*	0.764	0.365	0.000	0.000	0.077	0.839	–										
8. DASS-21 Depression		−0.129	−0.200^*^	0.557^**^	0.519^**^	0.437^**^	−0.147	0.478^**^	–									
	*p*	0.189	0.041	0.000	0.000	0.000	0.135	0.000	–									
9. DASS-21 Anxiety		−0.155	−0.300^**^	0.536^**^	0.530^**^	0.463^**^	−.0.16^*^	0.387^**^	0.695^**^	–								
	*p*	0.113	0.002	0.000	0.000	0.000	0.027	0.000	0.000	–								
10. DASS-21 Stress		−0.289^**^	−0.279^**^	0.566^**^	0.516^**^	0.543^**^	−0.083	0.382^**^	0.789^**^	0.747^**^	–							
	*p*	0.003	0.004	0.000	0.000	0.000	0.403	0.000	0.000	0.000	–							
11. Understanding/comm		−0.041	−0.091	0.376^**^	0.218^*^	0.214^*^	−0.237^*^	0.254^**^	0.423^**^	0.537^**^	0.456^**^	–						
	*p*	0.676	0.358	0.000	0.025	0.028	0.015	0.009	0.000	0.000	0.000	–						
12. Getting around		0.171	−0.053	0.306^**^	0.280^**^	0.208^*^	−0.344^**^	0.197^*^	0.375^**^	0.523^**^	0.338^**^	0.662^**^	–					
	*p*	0.081	0.590	0.002	0.004	0.033	0.000	0.044	0.000	0.000	0.000	0.000	–					
13. Self-care		0.082	−0.024	0.090	0.078	0.042	−0.204^*^	114	0.190	0.282^**^	0.176	0.624^**^	0.652^**^	–				
	*p*	0.407	0.811	0.360	0.431	0.668	0.036	0.245	0.052	0.004	0.072	0.000	0.000	–				
14. Getting along with people		−060	−083	196^*^	0.120	0.244^*^	−0.201^*^	0.070	0.234^*^	0.431^**^	0.320^**^	0.692^**^	0.613^**^	0.771^**^	–			
	*p*	0.541	0.398	0.045	0.224	0.012	0.040	0.475	0.016	0.000	0.001	0.000	0.000	0.000	–			
15. Life activities		−0.031	−0.179	0.248^*^	0.336^**^	0.144	−0.233^*^	0.286^**^	0.397^**^	0.485^**^	0.383^**^	0.683^**^	0.662^**^	0.683^**^	642^**^	–		
	*p*	0.757	0.068	0.011	0.000	0.143	0.017	0.003	0.000	0.000	0.000	0.000	0.000	0.000	0.000	–		
16. Participation in society		0.153	−0.086	0.263^**^	0.347^**^	0.071	−0.190	0.275^**^	0.391^**^	0.482^**^	0.326^**^	0.563^**^	0.669^**^	0.557^**^	0.468^**^	0.726^**^	–	
	*p*	0.119	0.385	0.007	0.000	0.471	0.053	0.004	0.000	0.000	0.001	0.000	0.000	0.000	0.000	0.000	–	
17. WHODAS 2.0 total score		0.087	−0.073	0.262^**^	0.295^**^	0.147	−0.319^**^	0.245^*^	0.372^**^	0.526^**^	0.381^**^	0.826^**^	0.821^**^	0.841^**^	0.800^**^	0.867^**^	0.781^**^	–
	*p*	0.380	0.457	0.007	0.002	0.134	0.001	0.012	0.000	0.000	0.000	0.000	0.000	0.000	0.000	0.000	0.000	–

Multiple correlation analysis. *p< 0.05, **p< 0.01.

Model 1 (D01-Understanding and Communicating) followed a similar pattern, beginning with a non-significant Step 1 [*R*^2^ = 0.015, *F*_(5,99)_ = 0.294, *p* = 0.915]. The introduction of affective temperaments significantly improved the model [Δ*R*^2^ = 0.188, total *R*^2^ = 0.202, *F*_(10,94)_ = 2.383, *p* = 0.015], with hyperthymic temperament as the only significant predictor (*β* = −0.231, *p* = 0.030). The final step incorporating psychological distress measures yielded the most robust model [Δ*R*^2^ = 0.148, total *R*^2^ = 0.350, *F*_(13,91)_ = 3.774, *p* < 0.001], where anxiety became the main predictor (*β* = 0.398, *p* = 0.008). Model 2 (D02-Getting Around) was unique in showing significant predictors in Step 1 (*R*^2^ = 0.086), with age associated (*β* = 0.275, *p* = 0.044) and disease duration (*β* = 0.259, *p* = 0.039) positively associated with mobility difficulties. The addition of temperamental variables produced the largest R^2^ change across all models [Δ*R*^2^ = 0.245, total *R*^2^ = 0.330, *F*_(10,94)_ = 4.640, *p* < 0.001], with hyperthymic temperament showing the strongest protective effect (*β* = −0.344, *p* = 0.001). The final model achieved the highest explanatory power among all domains [total *R*^2^ = 0.422, *F*_(13,91)_ = 5.118, *p* < 0.001], with both hyperthymic temperament (*β* = −0.258, *p* = 0.007) and anxiety (*β* = 0.394, *p* = 0.006) maintaining significance, indicating that mobility functioning is influenced by both temperamental resilience and psychological distress.

Model 3 (D03-Self-Care) did not achieve statistical significance at any step. The Step 1 was non-significant [*R*^2^ = 0.037, *F*_(5,99)_ = 0.753, *p* = 0.586], and the addition of temperamental variables provided minimal improvement [Δ*R*^2^ = 0.056, total *R*^2^ = 0.092, *F*_(10,94)_ = 0.955, *p* = 0.488]. The complete model including DASS-21 scales remained non-significant [total *R*^2^ = 0.155, *F*_(13,91)_ = 1.288, *p* = 0.235], suggesting that self-care functioning may be relatively independent of the affective temperaments and psychological distress variables examined in this study. Model 4 (D04-Getting Along with People) exhibited a unique pattern where neither Step 1 variables [*R*^2^ = 0.011, *F*_(5,99)_ = 0.215, *p* = 0.956] nor the addition of temperamental variables [total *R*^2^ = 0.128, *F*_(10,94)_ = 1.377, *p* = 0.203] were significant. However, with the incorporation of DASS-21 scales the model reached statistical significance [total *R*^2^ = 0.246, *F*_(13,91)_ = 2.287, *p* = 0.012], with anxiety as a highly significant predictor (*β* = 0.462, *p* = 0.005). This pattern suggests that interpersonal difficulties are primarily driven by current psychological distress rather than stable temperamental characteristics. Model 5 (D05-Life Activities) showed Step 1 significance (*R*^2^ = 0.050) with disease duration as a weak predictor (*β* = −0.259, *p* = 0.043). The temperamental variables significantly enhanced the model [Δ*R*^2^ = 0.176, total *R*^2^ = 0.226, *F*_(10,94)_ = 2.741, *p* = 0.005], with hyperthymic temperament as the primary predictor (*β* = −0.264, *p* = 0.012). The final Step 3L maintained significance [total *R*^2^ = 0.305, *F*_(13,91)_ = 3.076, *p* = 0.001], with anxiety becoming the sole significant predictor (*β* = 0.307, *p* = 0.047), indicating a shift from temperamental to symptom-based prediction of life activity limitations. Model 6 (D06-Participation in Society) began with significant Step 1 predictors (*R*^2^ = 0.080), including positive associations with age (*β* = 0.321, *p* = 0.019) and with disease duration (*β* = 0.300, *p* = 0.018). The temperamental variables substantially improved the model [Δ*R*^2^ = 0.186, total *R*^2^ = 0.266, *F*_(10,94)_ = 3.414, *p* = 0.001], with depressive temperament as a significant positive predictor (*β* = 0.261, *p* = 0.018). The final Step 3 achieved strong predictive validity [total *R*^2^ = 0.369, *F*_(13,91)_ = 4.100, *p* < 0.001], with anxiety becoming the main predictor (*β* = 0.385, *p* = 0.010).

Across all models, anxiety consistently emerged as the most robust predictor in final models, showing significant positive associations with six of the seven functional domains, while hyperthymic temperament demonstrated consistent protective. Results are shown in Table 3.

**Table 3:** Hierarchical regression models predicting WHODAS 2.0 disability domains (D01: Cognition, D02: Mobility, D03: Self-care, D04: Getting along, D05: Life activities, D06: Participation) and total disability scores. Step 1 (Demographics variables/Disease duration); step 2 (Temperament traits assessed with the TEMPS-A); step 3 (psychiatric comorbidities, assessed with the DASS-21). WHODAS 2.0 = World Health Organization Disability Assessment Schedule 2.0; TEMPS-A = Temperament Evaluation of Memphis, Pisa, Paris, and San Diego Auto-questionnaire; DASS-21 = Depression, Anxiety, and Stress Scales-21.

	D01	D02	D03	D04	D05	D06	Total
Step 1: Demographic variables and disease duration	β	β	β	β	β	β	β
Sex (female)	−0.004	−0.074	−0.043	−0.054	0.057	−0.033	−0.004
Age (years)	−0.01	0.275*	0.085	−0.029	0.124	0.321*	0.198
Education (years)	−0.023	−0.048	−0.092	0.025	−0.008	−0.011	−0.055
Relationship (partnered)	0.078	0.092	0.113	0.023	0.062	0.031	0.022
Disease duration (years)	−0.101	0.259*	−0.112	−0.076	0.26*	0.3*	−0.207
*R^2^*	0.015	0.086	0.037	0.011	0.05	0.08	0.038
*F*	0.294	1.856	0.753	0.215	1.046	1.724	0.782
Step2: Temperament traits							
Cyclothymic	0.197	0.104	−0.057	0.004	−0.028	0.12	0.023
Depressive	0.025	0.122	0.013	−0.038	0.197	0.261	0.14
Irritable	0.106	0.184	0.082	0.228	0.049	−0.074	0.085
Hyperthymic	−0.231*	−0.344**	−0.234*	−0.260*	−0.264*	−0.163*	−0.35**
Anxious	0.161	0.083	0.122	0.072	0.238	0.178	0.213
*R^2^*	0.202	0.33	0.092	0.128	0.226	0,266	0.255
Δ*R*^2^	0.188	0.245	0.056	0.117	0.176	0.186	0.217
*F*	2.383*	4.64**	0.955	1.377	2.741**	3.414**	3.211**
Step 3: Psychiatric comorbidity							
Depression	0.013	0.041	−0.021	−0.127	0.087	0.165	−0.037
Anxiety	0.398*	0.394**	0.255	0.462**	0.307*	0.385*	0.409**
Stress	0.231	0.042	0.179	0.149	0.082	−0.024	0.191
*R^2^*	0.35	0.422	0.155	0.246	0.305	0.369	0.379
Δ*R*^2^	0.148	0.092	0.063	0.118	0.079	0.103	0.125
*F*	3.774*	5.118**	1.288	2.287*	3.076**	4.1**	4.278**

*p< 0.05, **p< 0.01.

## 4 Discussion

In this study, we investigated disability assessed with WHODAS 2.0 in RRMS by analyzing its connection to psychological distress and emotional temperament traits. Age was significantly associated with increased mobility deficits and greater impairment in social participation. Our findings demonstrate that psychological factors, particularly temperament traits and psychiatric comorbidities (anxiety), significantly contribute to disability prediction beyond traditional demographic and clinical variables.

The initial model (Step 1) examining demographic variables and disease duration revealed limited predictive value, with age and disease duration emerging as the most consistent predictors across disability domains. Age has been consistently identified as a significant predictor of long-term disability in RRMS, which aligns with our findings showing positive associations between age and several disability dimensions (D02, D06). The significant positive correlation between disease duration and disability measures (D02, D05, D06) corroborates existing literature demonstrating the progressive nature of MS-related impairment over time impacting their ability to participate in social activities (39, 40). Also, studies of the MSFC which evaluates neurological impairment through arm function, walking ability, and cognitive processing, revealed that age-related factors increase mobility impairments, leading to social isolation (3). Both the EDSS and MSFC scores typically worsen with age, leading to reduced physical mobility, decreased social interactions, and increased dependency on support systems (41). Our results are consistent with studies that have described a correlation between age and mobility using WHODAS 2.0 (42, 43). On the contrary, sex, education level, and relationship status showed minimal predictive value in our cohort, suggesting that these demographic factors may have less influence on disability outcomes assessed with the WHODAS than previously assumed in some studies (44).

Furthermore, specific temperamental dimensions tested with the TEMPS-A have been associated with psychological distress, depression, anxiety, and stress associated with neuropsychiatric diseases (45). These e temperaments assessment reveals distinct correlations with diseases outcomes. The hyperthymic temperamental profile demonstrates significant associations with favorable clinical trajectories (46, 47). The underlying mechanisms appear to involve enhanced stress-response and psychological resilience (48). Conversely, cyclothymic, anxious, and depressive temperamental profiles correlate with increased disability and demonstrate strong associations with affective symptomatology (49) and impaired stress-response mechanisms (50). Previous studies investigating affective temperaments assessed with the TEMPS-A in RRMS are limited (31) and have highlighted their association with quality of life (51). Our findings in person with RRMS expand these observations, further demonstrating that hyperthymic temperament traits are associated with improved mobility, enhanced social engagement, and reduced overall disability levels, as measured by the WHODAS. This inverse correlation suggests that hyperthymic temperament may serve as a protective neurobiological mechanism in MS, modulating disease progression, functional capacity, and social participation across multiple domains.

Our study indicated that the anxiety score strongly predicted disability in RRMS. Previous epidemiological research has identified a prevalence of anxiety disorders in RRMS ranging from 15.8 to 57%, which is higher than rates found in the general population (52, 53). Among the various anxiety disorders present in MS patients, Generalized Anxiety Disorder is the most common, with a prevalence of 18.6%. Specific phobias and panic disorder occur at lower rates of 10.8 and 10.0%, respectively (54). Research shows anxiety and depression are often comorbid, with each condition increasing the risk of developing the other over time (55, 56). Our findings align with previous studies demonstrating how anxiety contributes to disability. For instance, anxiety may contribute to disease progression through stress-induced immunological activation (57–62). In our study, we found that anxiety s, rather than depression, were more significant predictors of disability as measured by the WHODAS. While previous research using the EDSS found links between depression and disability in persons with MS, the results vary across studies. This may be due to differences between the WHODAS and EDSS assessments, since EDSS primarily emphasizes physical impairment and mobility while WHODAS assesses multiple domains of functioning (58, 63, 64). Consequently, the greater predictive strength of anxiety and stress observed in our research may indicate WHODAS’s enhanced sensitivity to the psychological factors influencing daily functioning and social participation. This aligns with previous literature indicating that traditional disability measures like the EDSS may underestimate the impact of psychological symptoms on the overall functioning of persons with MS. On the contrary, a longitudinal study assessing disability progression using the WHODAS 2.0 in persons with MS showed that depression was associated with higher disability (65). Although the study included a large population of persons with MS, potential methodological limitations and the significant variability in baseline disability values make it necessary to confirm the findings in future studies. Thus, the heterogeneity of results previously observed in studies using the EDSS is also evident when using the WHODAS. The heterogeneity across studies may be linked to several factors: (1) differences in sample characteristics, particularly regarding depression severity and disease duration; (2) varying adjustment for potential mediators such as fatigue, cognitive impairment, or pain that may account for depression’s apparent effects; and (3) measurement differences in both depression (diagnostic criteria vs. symptom scales) and disability (WHODAS total score vs. specific subdomains). It is also possible that these conflicting results may be due to the contribution of multiple variables, both cognitive and psychological (65). In our study, the use of a homogeneous population without significant cognitive deficits represents a novelty in the literature. A multicenter study with a larger population is currently being planned to confirm our results.

Our study has a few limitations. First, it utilizes a cross-sectional design, which does not allow for discussing causal links or temporal sequences between psychological symptoms and functional outcomes. In addition, the research was performed at a single site. Moreover, we did not include in our cohort MS participants with a significant cognitive impairment. We used a screening cognitive assessment tool (MoCA) to minimize participant burden and avoid a lengthy study. Although MoCA is an effective screening instrument, it may overlook minimal or domain-specific cognitive deficits. Thus, future studies should employ more comprehensive cognitive batteries to better evaluate the full spectrum of cognitive function and their association with disability assessed with the WHODAS 2.0 scale. In addition, this study’s sample size of 105 participants, while reasonable (66), may limit the detection of smaller effects given the predictors analyzed. The risk of overfitting and unmeasured confounding variables should be considered when interpreting results. Future studies with larger, more diverse samples could strengthen these observations. While this study originally planned to analyze only the total WHODAS score, we expanded our model to include all WHODAS dimensions due to their consistent alignment with existing literature and recurrent predictor patterns. However, these additional findings should be interpreted cautiously as exploratory, serving as pilot data for future larger-scale validation.

## 5 Conclusion

This study found significant associations between psychological factors (particularly anxiety) and higher disability assessed with the WHODAS 2.0 in RRMS, while hyperthymic temperament correlated with lower disability. As a cross-sectional analysis, these findings suggest clinical relevance of psychological assessment in MS care, but causal relationships remain unclear. Future longitudinal studies should investigate these interactions over time.

## Data Availability

The raw data supporting the conclusions of this article will be made available by the authors, without undue reservation.
